# Molecular Analysis of the *ABCA4* Gene Mutations in Patients with Stargardt Disease Using Human Hair Follicles

**DOI:** 10.3390/ijms21103430

**Published:** 2020-05-13

**Authors:** Aneta Ścieżyńska, Marta Soszyńska, Michał Komorowski, Anna Podgórska, Natalia Krześniak, Aleksandra Nogowska, Martyna Smolińska, Kamil Szulborski, Jacek P. Szaflik, Bartłomiej Noszczyk, Monika Ołdak, Jacek Malejczyk

**Affiliations:** 1 Department of Histology and Embryology, Medical University of Warsaw, 02-004 Warsaw, Poland; asciezynska@wum.edu.pl (A.Ś.); nogowskaaleksandra@gmail.com (A.N.); martyna.wroblewska90@gmail.com (M.S.); monika.oldak@wum.edu.pl (M.O.); 2 Laboratory of Experimental Immunology, Military Institute of Hygiene and Epidemiology, 01-163 Warsaw, Poland; marta.soszynska@onet.eu (M.S.); michalpiotrkomorowski91@gmail.com (M.K.); 3 Center for Preclinical Research and Technology, Medical University of Warsaw, 02-097 Warsaw, Poland; 4 Molecular Biology Laboratory, Department of Medical Biology, Cardinal Stefan Wyszyński Institute of Cardiology, 04-628 Warsaw, Poland; apodgorska@ikard.pl; 5 Department of Plastic Surgery, Medical Centre for Postgraduate Education, 00-416 Warsaw, Poland; natalia.krzesniak@wp.pl (N.K.); bnoszczyk@gmail.com (B.N.); 6 Department of Ophthalmology, Medical University of Warsaw, 03-709 Warsaw, Poland; donbors@yahoo.com (K.S.); jacek@szaflik.pl (J.P.S.)

**Keywords:** hair follicles, *ABCA4* gene mutations, Stargardt disease, *ABCA4* retinopathies, molecular analysis, splice-site variants

## Abstract

*ABCA4* gene mutations are the cause of a spectrum of *ABCA4* retinopathies, and the most common juvenile macular degeneration is called Stargardt disease. *ABCA4* has previously been observed almost exclusively in the retina. Therefore, studying the functional consequences of *ABCA4* variants has required advanced molecular analysis techniques. The aim of the present study was to evaluate whether human hair follicles may be used for molecular analysis of the *ABCA4* gene splice-site variants in patients with *ABCA4* retinopathies. We assessed *ABCA4* expression in hair follicles and skin at mRNA and protein levels by means of real-time PCR and Western blot analyses, respectively. We performed cDNA sequencing to reveal the presence of full-length *ABCA4* transcripts and analyzed *ABCA4* transcripts from three patients with Stargardt disease carrying different splice-site *ABCA4* variants: c.5312+1G>A, c.5312+2T>G and c.5836-3C>A. cDNA analysis revealed that c.5312+1G>A, c.5312+2T>G variants led to the skipping of exon 37, and the c.5836-3C>A variant resulted in the insertion of 30 nucleotides into the transcript. Our results strongly argue for the use of hair follicles as a model for the molecular analysis of the pathogenicity of *ABCA4* variants in patients with *ABCA4* retinopathies.

## 1. Introduction

The *ABCA4* gene (OMIM 601691; GenBank:NG_009073.1) encodes an ATP-binding cassette transporter which translocates retinoid intermediates of the visual cycle across the disc membranes of photoreceptor cells [1]. Mutations of the *ABCA4* gene are the cause of more than 95% of cases of Stargardt disease (STGD)—the most common form of inherited juvenile macular degeneration—as well as other monogenic retinal diseases, the so-called *ABCA4* retinopathies [2].

Some of the *ABCA4* mutations frequently occur in certain ethnic groups, e.g., European [3,4,5,6,7,8], Brazilian [9], Mexican [10] and South African [11] populations. According to the Human Gene Mutation Database (HGMD Professional version 2019.4), 1467 *ABCA4* gene mutations have been identified so far, though novel, potentially pathogenic *ABCA4* gene variants are still being detected. The *ABCA4* gene carries a high number of non-canonical splice variants and protein-truncating mutations, which constitute the second highest type of genetic aberration, after missense mutations [12,13]. Residual activity of the mutant ABCA4 protein supposedly determines the severity of the disease [14]. Unfortunately, to date, the pathogenicity of many *ABCA4* variants remains unclear, and some supposedly deleterious variants may influence the onset of Stargardt disease in different ways [15]. Additionally, some variants called “extremely” hypomorphic and modifier alleles, may result in different phenotypes when residing in cis or in trans with other pathogenic variants [16,17,18,19] as described in detail in the most recent review of Cremers et al. [20]. Therefore, identification of the *ABCA4* mutations, as well as assessment of their pathogenicity, is essential for affected families and may be helpful in prediction of the disease’s severity and the introduction of precautions which may reduce the disease’s progression [21,22].

Thus far, functional studies have been limited due to difficulties in the development of functional assays to investigate the effects of *ABCA4* mutations. The biological effect of mutations may differ from those predicted on the basis of bioinformatical analyses, therefore, the assessment of novel, rare, non-canonical splice-site variants cannot rely fully on in silico computations [23]. Assessment of the effects of non-canonical splice-site variants with the use of mini- or midigenes [24] requires an advanced, complex methodology [25], which may be expensive and laborious. Although the effects of *ABCA4* splice-site variants have been evaluated in in vitro assays [16,24,26,27,28,29,30], these may not always mimic splice defects in vivo [24]. The analysis of deep intronic variants with induced pluripotent stem cell-derived photoreceptor cells (iPSC-derived PCs) is an even more tedious task [27,31,32,33]. Therefore, novel methods for assessment of *ABCA4* gene mutations are highly desirable.

*ABCA4* gene expression appears to be mostly retina-specific [34], which has made the assessment of the biological role of different *ABCA4* variants almost impossible. Interestingly, a comprehensive analysis of *ABCA4* expression levels in a broad range of tissues [35] revealed the presence of the gene in the epididymis, duodenum, small intestine and kidney. Recent findings show that the *ABCA4* gene may also be expressed in human skin and hair follicles [36,37].

Analysis of RNA isolated from cultured human keratinocytes or dermal fibroblasts has revealed *ABCA4* transcript alterations caused by splice-site mutations located mainly in the introns of the latter half of the *ABCA4* gene [16,23,26,27,29,31,33,38]. It has been reported that normal human keratinocytes express an alternatively spliced truncated 70 kDa isoform of *ABCA4* [36]. Nevertheless, knowledge about the presence of the full-length *ABCA4* transcripts in human skin cells and hair follicles is incomplete and requires elucidation. Therefore, the present study investigated the presence and expression of the *ABCA4* full-length transcript in human hair follicles and skin, in the context of their possible application for the molecular evaluation of *ABCA4* splice-site variants. Additionally, using hair follicles from patients with Stargardt disease, we investigated the molecular consequences of selected *ABCA4* variants on gene processing.

## 2. Results

### 2.1. ABCA4 Gene is Expressed in Human Hair Follicles and Skin

**Figure 1:**
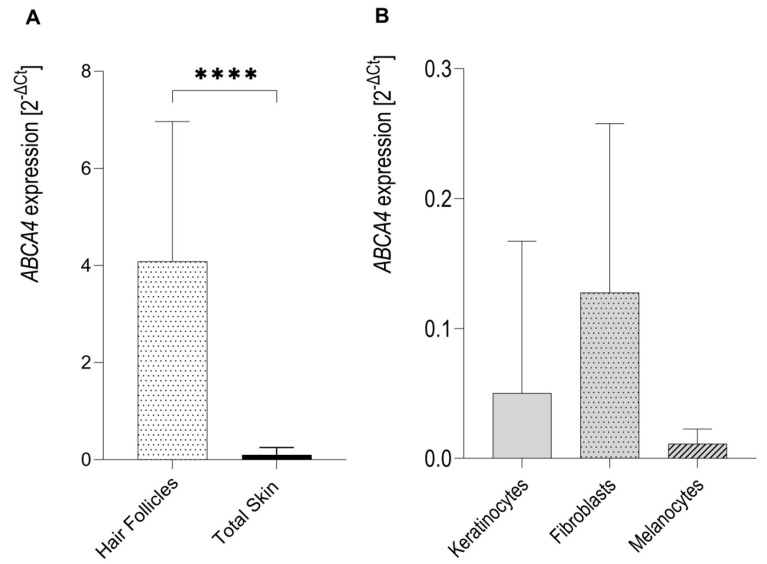
Expression of the *ABCA4* mRNA in hair follicles, skin lysates, as well as keratinocytes, dermal fibroblasts and melanocytes as measured by qRT-PCR for TaqMan probe Hs00979586_m1 (*ABCA4* exons 35–36). (**A**) Expression of the *ABCA4* gene in the isolated hair follicles was significantly higher than in total skin lysates. (**B**) Relatively very low expression of the *ABCA4* gene was detected in fibroblasts, keratinocytes and melanocytes. ∗∗∗∗ *p* < 0.0001 as calculated by Mann-Whitney *U* test.

The *ABCA4* gene was found to be expressed in human eyebrow hair follicles (*n* = 8) and skin explants (*n* = 8) (Figure 1A). Expression of the *ABCA4* gene in hair follicles was over fifty-fold higher than in the skin (*p*-value < 0.0001 by Mann-Whitney *U* test). Some, but relatively very few, *ABCA4* expressions were also detected in cultured human primary keratinocytes (*n* = 8), dermal fibroblasts (*n* = 8) and melanocytes (*n* = 8) (Figure 1B). There were no statistically significant differences in *ABCA4* mRNA expression between keratinocytes, fibroblasts and melanocytes (*p*-value = 0.1562 by one-way ANOVA).

Additional evaluation of the particular *ABCA4* exons in hair follicles and total skin showed that expression of exons 3–4 was lower as compared to the other exons (Table 1).

### 2.2. Hair Follicles and Skin Cells Express the Full-Length *ABCA4* Transcript and Protein

**Figure 2:**
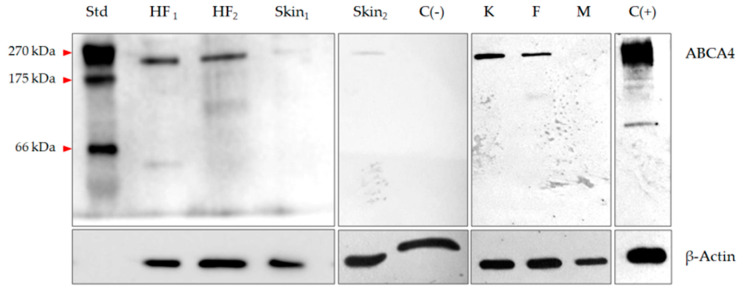
Expression of the *ABCA4* gene in hair follicles, skin cells and skin lysates measured at the protein level with Western blot assay. HF, hair follicle; Skin, skin sample positive for *ABCA4* mRNA; K, keratinocytes; F, fibroblasts; M, melanocytes; C(+), retinoblastoma lysate; C(–), skin sample negative for *ABCA4* mRNA; Std, broad-range protein standard.

The presence of all *ABCA4* exons was confirmed in the analyzed hair follicle samples (*n* = 4), cultured keratinocytes (*n* = 4), fibroblasts (*n* = 4) and melanocytes (*n* = 4) by means of Sanger sequencing. Western blot analysis of hair follicles, skin protein extracts and cultured skin cells confirmed the presence of the main *ABCA4* immunoreactive band, corresponding to a molecular mass of 250 kDa. In one skin sample, expression of the *ABCA4* gene was not detected and this sample was used as a negative control. The result of the Western blot analysis of representative hair follicles and skin samples is presented in Figure 2.

### 2.3. Analysis of the *ABCA4* Splice-Site Variants in Hair Follicles

Considering that the expression of the *ABCA4* gene in the skin samples was significantly lower than in the hair follicles, we used the latter for further experiments aimed at assessing the sequence of *ABCA4* transcripts in patients’ samples.

**Figure 3:**
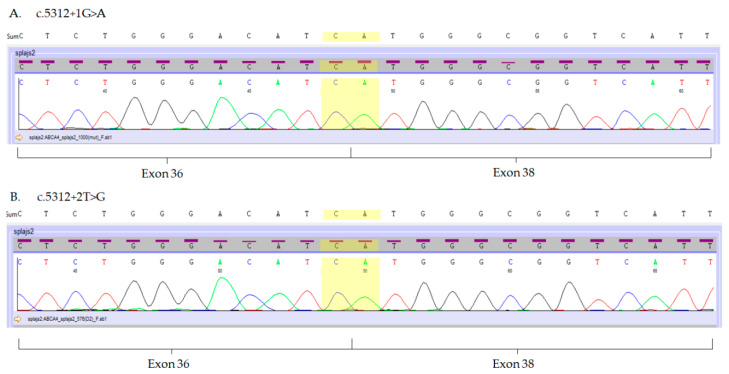

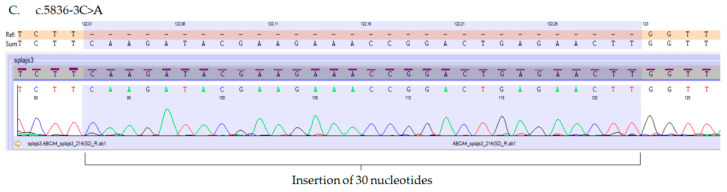
Electropherograms of the mutated *ABCA4* transcripts isolated from hair follicles. (**A**,**B**) Mutations c.5312+1G>A and c.5312+2T>G result in the exon 37 skipping. Boundaries of exon 36 and 38 are marked in yellow. (**C**) Mutation c.5836-3C>A results in an insertion of 30 nucleotides (marked purple) into the *ABCA4* transcript between the 41st and 42nd exon of the *ABCA4* transcript.

In order to evaluate the usefulness of eyebrow hair follicles as a tool for the molecular analysis of *ABCA4* variants, we investigated the consequences of the three *ABCA4* splice-site variants c.5312+1G>A, c.5312+2T>G and c.5836-3C>A, previously detected in patients with Stargardt dystrophy [7]. In those patients, *ABCA4* gene mutations were found in a compound heterozygous state with missense (NM_000350.2:c.[1622T>C;3113C>T], NP_000341.2:p.[Leu541Pro;Ala1038Val] or NM_000350.2:c.5882G>A, NP_000341.2:p.Gly1961Glu) or nonsense (NM_000350.2:c.4872G>A, NP_000341.2:p.Trp1624*) *ABCA4* mutations [7]. Direct Sanger sequencing revealed that variants c.5312+1G>A and c.5312+2T>G result in the skipping of exon 37 (Figure 3A,B), whereas mutation c.5836-3C>A leads to the insertion of thirty nucleotides of the intron adjacent to exon 42 (Figure 3C).

### 2.4. Analysis of the ABCA4 Gene Expression in Hair Follicles of Patients with Stargardt Disease

**Figure 4:**
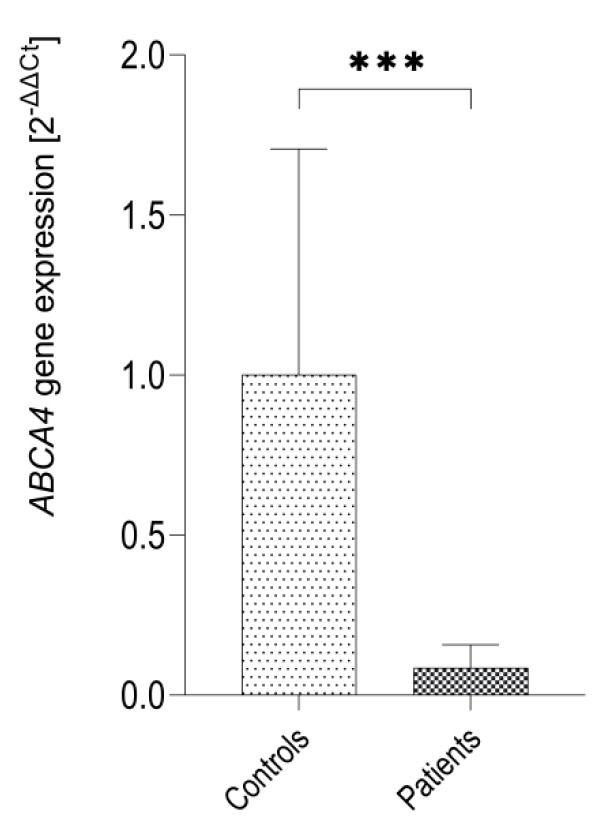
Expression of the *ABCA4* gene in hair follicles (HF) of patients with Stargardt disease (*n* = 3) and healthy controls (*n* = 8) as measured by qRT-PCR for TaqMan probe Hs00979586_m1 (*ABCA4* exons 35–36). ∗∗∗ *p* = 0.0001 as evaluated by Mann-Whitney *U* test.

In order to analyze the overall expression of the *ABCA4* gene expression in patients with Stargardt disease we performed qRT-PCR. The results show that *ABCA4* mRNA is also expressed in the hair follicles obtained from patients with Stargardt disease, however, this expression was significantly lower than in controls (*p* = 0.0001 by Mann-Whitney *U* test) (Figure 4).

## 3. Discussion

Here, we report that full-length *ABCA4* transcript is highly expressed in human eyebrow hair follicles and may be thus considered useful for the molecular analysis of splice-site *ABCA4* mutation consequences in patients with *ABCA4* retinopathies. The applicability of the hair follicle model was confirmed by the analysis of three different splice-site *ABCA4* variants from patients with Stargardt disease.

The *ABCA4* gene was previously thought to be expressed at very low levels in non-ocular tissues. Although a previous high-throughput mRNA analysis of ABC transporters failed to detect *ABCA4* expression in human total skin [39], nevertheless, we detected low levels of *ABCA4* expression in human skin explants. On the other hand, a relatively high *ABCA4* gene expression was observed in human hair follicles. This is in agreement with the observation made by Haslam et al., who also reported a high expression level of the *ABCA4* gene throughout the epithelium of hair follicles [37]. Interestingly, we found that mRNA expression of the *ABCA4* exons 3–4 was considerably lower as compared to other exons. This may implicate presence of alternate splicing in normal hair follicles, though one cannot exclude the possibility of an insufficient qPCR reaction for this particular probe. Nevertheless, we confirmed the presence of all *ABCA4* exons of transcripts isolated from human hair follicles, cultured human keratinocytes, fibroblasts and melanocytes by means of direct Sanger sequencing. Presence of the *ABCA4* full-length protein was also confirmed by the Western blot method.

In the hair follicles obtained from healthy controls, a high variability of the *ABCA4* gene expression was observed. This was probably caused by individual differences of the *ABCA4* gene expression in the population. However, it may be also associated with an unknown function of the *ABCA4* in the hair follicles. It is also tempting to speculate that the slightly decreased expression of the *ABCA4* gene in the control group (though significantly greater than that observed in the patients) may be related to existence of the pathogenic *ABCA4* variants, as has been demonstrated in a canine model of Stargardt disease [40]. This, however, requires further analysis of *ABCA4* gene expression in the probands’ parents.

Interestingly, *ABCA4* mRNA expression in keratinocytes was very low compared to hair follicles, yet ABCA4 protein was detected at a relatively high level by the Western blot method. This difference may be due to variability between samples of different origins. However, this phenomenon may be also explained by the posttranscriptional regulation of the ABCA4 transporter in the skin, as has been observed for other ABCA transporters in tissues in which protein expression was much higher that would be expected based on their level of mRNA expression [41].

Variants potentially affecting RNA splicing can be directly tested with the use of qRT-PCR on accessible cells (e.g., skin cells, lymphoblasts), providing that they constitutively express a sufficient level of the gene of interest. To assess whether hair follicles are a suitable source of material for the study of the consequences of *ABCA4* mutations, we analyzed hair follicle-derived mRNA obtained from patients with Stargardt disease carrying representative c.5312+1G>A, c.5312+2T>G and c.5836-3C>A *ABCA4* variants [7]. Our findings are in concordance with the results of Schulz et al. who also observed skipping of the 37th exon caused by the c.5312+1G>A mutation using in vitro splicing assay [42]. Furthermore, our results concerning c.5836-3C>A mutation were also consistent with the observations of Sangermano et al. by means of midigene assays [24]. It would also be of great interest to analyze other *ABCA4* gene splice variants, especially those located at the 5′ region of the *ABCA4* gene. Unfortunately, due to lack of appropriate clinical material from patients, analysis of such variants could not be performed in the present study and awaits further investigation.

Analysis of the overall *ABCA4* gene expression in patients with Stargardt disease showed a high reduction as compared to healthy controls. This is in agreement with results obtained from different cell types, which also showed a reduction of normally spliced full-length *ABCA4* mRNA in fibroblasts of patients with Stargardt disease [23]. It needs to be further elucidated whether the expression level of the *ABCA4* gene in patients with Stargardt disease correlates with disease severity.

Eyebrow hair follicles are regularly plucked by millions of women worldwide. Unlike a skin biopsy, no anesthetic is needed and no skin injuries are inflicted upon the patient. In addition, material can be obtained directly from patients and their families and sent to the laboratory without the need of patient arrival. About 10–15 eyebrow hair follicles yield an amount of RNA sufficient to conduct a preliminary molecular assessment of *ABCA4* variant pathogenicity and *ABCA4* gene expression analysis. This method appears to be simple and inexpensive, especially as it does not require cell culture or the highly advanced generation of mini-, midigenes or photoreceptor precursor cells (PPCs).

It should be stressed, however, that not all molecular and functional analyses can be performed on non-cultured cells from hair follicles. It is impossible to block nonsense-mediated decay in non-cultured hair follicle cells, therefore *ABCA4* splice-site variant consequences can be missed in heterozygous patients. Also, reverse transcription reactions are difficult to conduct on large genes where 5′ of the gene may not be efficiently retrotranscribed, as compared with 3′ of the gene. For this purpose, the construction of mini- or midigenes is more favorable, even though mini-genes may result in the creation of splicing artefacts [13]. In order to improve the accuracy of these assays, interesting results regarding functional analysis of the *ABCA4* splice-site variants were obtained with photoreceptor precursor cells generated from skin fibroblasts [24,27,32,33]. Still, the invasiveness of skin biopsies may impede these analyses. Hair follicles may be non-invasively obtained from patients multiple times and may also be a source of cultured epithelial stem cells [43]. Whether consequences of the *ABCA4* splice-site variants in cultured hair follicle cells would lead to similar outcomes as those observed in PPCs needs further investigation.

In conclusion, analysis of mRNA from hair follicles appears to be a reliable method to assess the biological consequences of functional changes of the *ABCA4* gene in patients with Stargardt disease and possibly other *ABCA4* retinopathies. Furthermore, this method is non-invasive for the patients and is relatively simple and inexpensive as compared to other methods based on highly advanced molecular techniques.

## 4. Materials and Methods

### 4.1. Patients and Controls

Skin biopsies were acquired from eight healthy individuals subjected to aesthetic breast reduction or aesthetic abdominoplasty in the Department of Plastic Surgery, Medical Centre of Postgraduate Education, Orlowski Memorial Hospital in Warsaw. Up to 30 hair follicles were plucked with tweezers from eyebrows of eight healthy volunteers and three patients with Stargardt disease bearing splice-site *ABCA4* gene mutations (NM_000350.3:c.5312+1G>A, NM_000350.3:c.5312+2T>G or NM_000350.3:c.5836-3C>A) who were diagnosed in the SPKSO Ophthalmic University Hospital, Medical University of Warsaw. Written informed consent was obtained from all participants. The study was approved by the Ethics Committee of Medical University of Warsaw (KB/191/2010 from 21 September 2010) and the Ethics Committee at the Medical Centre of Postgraduate Education, Orlowski Memorial Hospital in Warsaw (63/PB/2016 from 16 November 2016).

### 4.2. Culture of Primary Keratinocytes, Fibroblasts and Melanocytes

Keratinocyte cultures were established as described previously with minor modifications [44]. Briefly, the epidermis was separated from the dermis with 2.5 U/mL of dispase by an overnight digestion at 4 °C. Keratinocytes and melanocytes were released from the epidermis after 5 min of cleavage in 0.25% trypsin at 37 °C with subsequent pipetting. The cells were cultured in Medium 254 (Thermo Fisher Scientific, Waltham, MA, USA) supplemented with Human Melanocyte Growth Supplement-2, PMA-free (Thermo Fisher Scientific, Waltham, MA, USA). Following one-week culture, melanocytes were separated from keratinocytes with a short (up to 2 min) trypsinization. Keratinocytes were subsequently cultured in Keratinocyte Growth Medium (PromoCell, Heidelberg, Germany) supplemented with Keratinocyte Supplement Mix (PromoCell, Heidelberg, Germany). Dermal fibroblasts were released by 0.6 U/mL of Collagenase (Serva Electrophoresis GMbH, Heidelberg, Germany) digestion for 1 h at 37 °C with subsequent pipetting. Then they were cultured in DMEM (Thermo Fisher Scientific, Waltham, MA, USA) supplemented with 10% Fetal Bovine Serum (EURx, Gdansk, Poland). Cells in passages 1–2 were used in subsequent experiments.

### 4.3. RNA Isolation and Quantitative mRNA Measurements

Skin explants cut slightly below the basal layer of the epidermis with shallow fragments of the dermis and eyebrow hair follicles were submerged in the RNAlater Storage Solution (Thermo Fisher Scientific, Waltham, MA, USA) and stored at 4 °C prior to subsequent isolation of total RNA and protein within 6 h. Total RNA from skin explants, skin cells and hair follicles was extracted with NucleoSpin^®^ TriPrep (with rDNase digestion) (Macherey-Nagel, Dürren, Germany) and transcribed into cDNA with RevertAid First Strand cDNA Synthesis Kit (Thermo Fisher Scientific, Waltham, MA, USA). For quantitative PCR, 20 ng of cDNA per reaction was used. All real-time PCR reactions were performed on the ABI 7500 Fast Real-time PCR system (Applied Biosystems, Inc., Thermo Fisher Scientific, Waltham, MA, USA). Expression levels of the *ABCA4* gene in hair follicles, skin cells and skin explants from healthy donors and hair follicles from the patients were analyzed with Hs00979586_m1 TaqMan^®^ probe (Thermo Fisher Scientific, Waltham, MA, USA). TBP gene (TATA-box binding protein, Hs00427620_m1) was used as a reference gene for further normalizations. All samples were run in triplicates. The relative level of mRNA expression was calculated by the ΔCt or ΔΔCt method.

qRT-PCR evaluation of particular *ABCA4* exon mRNA expression in hair follicles and skin cells was performed with the use of TaqMan^®^ probes presented in Table 2. PCR reactions were performed as described above and specific exon expression was presented as number of cycle threshold (Ct).

### 4.4. Sanger Sequencing of the *ABCA4* Transcripts

PCR primers for the analysis of the full-length *ABCA4* transcript were designed with Primer 3 Plus software [45] based on the NM_000350.3 reference sequence. The primers were designed to encompass an area of the five to six adjacent exons, therefore the presence of the full-length *ABCA4* transcript was analyzed with 11 sets of primers, listed in Table 3.

PCR products were visualized on 2% agarose gel and excised with NucleoTrap^®^ (Macherey-Nagel, Dürren, Germany). In order to visualize PCR products of keratinocytes, fibroblasts and melanocytes, re-amplification for 35 cycles have been performed. Purified products were sequenced with BigDye Terminator v3.1 Cycle Sequencing Kit (Applied Biosystems, Thermo Fisher Scientific Inc.) according to the manufacturer’s instructions and subsequently sequenced on 3500xL Genetic Analyzer (Applied Biosystems, Thermo Fisher Scientific Inc.). The results were analyzed with Variant Reporter v.3.1. (Thermo Fisher Scientific Inc.).

### 4.5. Protein Extract Isolation and Western Blot Analysis

Proteins were extracted from the hair follicles and skin explants, as well as cultured keratinocytes, dermal fibroblasts and melanocytes, with NucleoSpin^®^ TriPrep (Macherey-Nagel), as described in detail by the manufacturer. Lysates were pelleted and resuspended in a buffer containing 150 mM NaCl, 1% Triton X-100, 0.5% sodium deoxycholate, 0.1% SDS and 50 mM Tris (pH 8.0). Protein concentration was evaluated with Pierce BCA Protein Assay Kit (Thermo Fisher Scientific Inc.) and 20 µg protein samples were separated by SDS–10% PAGE, transferred onto a nitrocellulose membrane (Thermo Fisher Scientific Inc.), blocked with Pierce Protein-Free Blocking Buffer (Thermo Fisher Scientific Inc.) and incubated at 4 °C overnight with 1:250 rabbit anti-*ABCA4* (cat. no. PA5-87983, Thermo Fisher Scientific Inc.) or 1:500 mouse anti-β-actin (cat. no. A1978, Sigma-Aldrich, St. Louis, MO, USA). Secondary HRP-conjugated goat anti-rabbit IgG antibodies (cat. no. GTX213110-01, GeneTex) or rabbit anti-mouse IgG antibodies (cat. no. GTX213111-01, GeneTex, CA, USA) were used and visualized with SuperSignal West Pico Chemiluminescent Substrate (Thermo Fisher Inc.). As an ABCA4 positive control, 20 µg of Y79 human retinoblastoma cell lysate (sc-2240, Santa Cruz Biotechnology) was used. A skin sample in which *ABCA4* mRNA expression was not detected by qRT-PCR served as a negative control. Broad range BlueEasy Prestained Protein Marker (Nippon Genetics Co., Dürren, Germany) was used as a protein standard.

### 4.6. Statistical Analysis

Statistical analyses and graphs were generated with GraphPad Prism 8 (GraphPad Software, San Diego, CA, USA). The nonparametric Mann Whitney *U* test and the one-way ANOVA were used to compare *ABCA4* expression levels in different studied groups. *p*-value < 0.05 was considered significant.

## Figures and Tables

**Table 1:** Average Ct of *ABCA4* gene expression in hair follicles, skin samples, keratinocytes, dermal fibroblasts and melanocytes.

Exon Number	3–4	10–11	20–21	29–30	35–36	40–41	49–50
Hair follicles (*n* = 8)	33.72	28.18	28.53	27.65	27.22	27.05	27.28
Total skin samples (*n* = 4)	37.77	32.51	33.17	Not tested	33.48	Not tested	31.92
Keratinocytes (*n* = 4)	35.07	34.42	34.15	Not tested	33.02	Not tested	34.12
Fibroblasts (*n* = 4)	33.67	32.75	32.54	Not tested	32.15	Not tested	31.48
Melanocytes (*n* = 4)	37.87	37.07	37.01	Not tested	36.29	Not tested	38.46

**Table 2:** Localization of the TaqMan probes within the *ABCA4* gene exons.

TaqMan^®^ Probes	Exon Boundary
Hs00979588_m1	3–4
Hs00979567_m1	10–11
Hs00979574_m1	20–21
Hs00979581_m1	29–30
Hs00979586_m1	35–36
Hs00979589_m1	40–41
Hs00979594_m1	49–50

**Table 3:** List of set of primers and annealing temperatures (T_a_) used during *ABCA4* transcript amplification.

*ABCA4* Exons	Forward Primer (5′–3′)	Reverse Primer (5′–3′)	Amplicon Length	T_a_
2–4	GGAACTCGTGTGGCCTTTATCTTTATT	GTCCATGAATTGGGACAAGATGTGTAG	331	58.2
4–8	AGCTACACATCTTGTCCCAATTCATGG	TGTCTTCATACCAGTTGAAGGAGAGCA	645	58.2
8–12	CTCGGGTGCTCTCCTTCAACTGGTAT	TGGTAGAGAGCTGGTCCAGGGATACAT	698	61.3
12–15	GTGCCCTCTCTCTACTGGAGGAAAACA	AGACTGGCCTTGGAGAAGAAGGTGCT	670	61.3
15–20	CACCTTCTTCTCCAAGGCCAGTCTG	CAGGATGTTGTGCTGTGGACACATG	787	61
20–25	CACAGCACAACATCCTGTTCCAC	GAAATTCCAAAACTGCTGAGACCAA	769	57.1
25–30	GGCTGACCTTGGTCTCAGCAGT	CCTGCAGGATGGTGAAGGGTTG	706	58.6
30–36	CACAGGTCAACCCTTCACCATCCTG	TTGGATTTGTTCACCCGCTCCTGGATCA	691	61
36–41	ATCTGCGTGATTTTCTCCATGTCCTT	ATCATCTTCATCAACAATGGGCTCCT	708	56.4
40–46	GAAGGGGTGGTGTACTTCCTCCTGAC	CCTTCTCTGATGATGCTCACGATGAC	707	62.7
44–49	GTCCATCAAAATATGGGCTACTGTCCT	CTGGTCCAGTGTGGTCTGTGTGACT	702	61
